# Osteomyelitis Secondary to Infected Cephalhematoma in a Female Neonate: A Case Report and Clinical Insights

**DOI:** 10.1155/crpe/5285153

**Published:** 2026-09-07

**Authors:** Mahnoor Khan, Nimra Rabbani, Yasser Masood, Gohar Javed, Jumana Aljbour

**Affiliations:** ^1^ Department of Pediatrics, Shifa College of Medicine, Islamabad, Pakistan; ^2^ Department of Pediatrics, Shifa International Hospital Ltd., Islamabad, Pakistan; ^3^ Department of Neurosurgery, Shifa International Hospital Ltd., Islamabad, Pakistan; ^4^ Department of Pediatrics, Islamic University of Gaza, Gaza, State of Palestine, iugaza.edu.ps

**Keywords:** cephalhematoma, infected cephalhematoma, neonate, osteomyelitis, pediatric neurosurgery, *Staphylococcus aureus*

## Abstract

Cephalhematomas are subperiosteal collections of blood resulting from birth trauma. Usually benign, they tend to resolve spontaneously within a few weeks; however, on rare occasions, they may develop an infection, which can lead to complications such as sepsis, meningitis, and osteomyelitis. Patients present with nonspecific symptoms, making it difficult and crucial to diagnose and treat them promptly. Across literature, there have been limited reports of infected cephalhematomas leading to osteomyelitis, and all such cases have mentioned the involvement of the parietal bone. We present the case of osteomyelitis secondary to an infected cephalhematoma in a female neonate, along with detailed insights into the diagnosis, treatment, and prognosis of the patient. This case highlights the importance of early recognition of the disease, which, in our patient, was first noted on the 5th day of life and properly diagnosed on the 29th day of life, and multidisciplinary treatment in preventing possibly fatal complications.

## 1. Introduction

A neonatal cephalhematoma is a subperiosteal hemorrhage, resulting in a localized collection of blood [1]. It is the most common birth‐related trauma occurring in approximately 1%‐2% of all deliveries, more frequently in those with instrumental assistance, but it has also been seen in cesarean sections [2].

Most cases resolve spontaneously without intervention in the first few weeks of life; however, larger lesions may require close observation. In rare circumstances, the lesions may acquire a secondary infection, which can lead to complications such as meningitis, osteomyelitis, and sepsis [3]. Here, we report the case of a full‐term female infant who developed an infected cephalhematoma on the 29th day of life, which was subsequently found to be associated with underlying osteomyelitis 10 days later—an uncommon but clinically significant finding. With only approximately 60 cases reported worldwide, this represents a rare clinical occurrence [4].

## 2. Case Presentation

The patient was a female infant born at full term via emergency lower‐segment cesarean section due to placental abruption at an outside hospital. The mother was G2P0 + 1, with a history of a previous ectopic pregnancy but no known history of maternal infections during this pregnancy. The APGAR scores at birth were not known; however, the patient was discharged uneventfully from the hospital.

On the fifth day of life, a swelling was noted at home on the patient’s left parietal region. Initially, the lesion measured around 2‐3 cm in diameter. By the 25th day, the lesion had grown to 6 cm in diameter, and she was thriving well. She was taken to a local hospital, where an ultrasound of the head was performed to ascertain the nature of the swelling. The scan revealed a collection of 25 mL of fluid over the left parietal region. She was diagnosed with a cephalhematoma, and the parents were counseled to simply observe the lesion and come back if it increases in size or the patient becomes unwell. Four days later, on the 29th day of life, they returned to the hospital with the lesion now measuring 7 cm. The swelling was drained using needle aspiration to remove 35 mL of pus. She was diagnosed with an infected cephalhematoma. The culture sensitivity report, days later, yielded methicillin‐susceptible Staphylococcus *aureus* (MSSA). Due to the increasing size of the cephalhematoma along with the presence of pus, a CT brain was performed to evaluate deeper involvement and possible complications. The CT report revealed a subgaleal hematoma of approximately 19 mL in the left parietal region along with evolving blood clots (acute on chronic pattern). Associated calvarial lucencies were also seen, which suggested pressure‐related modalities; however, the brain parenchyma was normal. The patient was diagnosed with an infected cephalhematoma and referred to a tertiary care hospital for further management.

The patient presented to the emergency department of our institution on the 31st day of life with a grossly visible swelling about 7.5 cm in diameter on the left parietal region. The patient weighed 3.5 kg, was vitally stable, afebrile, active, alert, tolerating oral feeds well, and had a normal systemic examination with no signs of infection or inflammation. Investigations revealed C‐reactive protein (CRP) 207 mg/L and white blood cell count (WBC) 18,290/μL; other baselines were normal (trends of the CRP and WBC are mentioned in Table 1). The blood culture did not yield any growth, which ruled out systemic infections. A CSF culture was not obtained due to the absence of fever or signs of meningeal irritation. A repeat CT scan was not performed due to financial constraints and the previous scan having been performed only 2 days prior. Incision and drainage was performed, which extracted 30 mL of pus (sent for culture sensitivity testing), and curettage of the obviously necrotic bone was performed. A bone biopsy was not deemed necessary because the causative organism had already been identified in the first culture, and the necrotic bone had been identified intraoperatively and managed with curettage. A subgaleal drain was placed, and the patient was shifted to the NICU.

**TABLE 1:** Table showing trends of CRP and white blood cell count during the patient’s course of treatment.

Timeline	CRP levels (mg/L)	White blood cell count (/μL)
1st day of admission (31st day of life)	207	18920
1st postoperative day (32nd day of life)	196	19350
Discharge (POD # 5) (36th day of life)	16.70	20010
2nd readmission (39th day of life)	5.36	23260
Day of discharge (POD # 8 after 2nd operation) (48th day of life)	4.54	10780
1‐month follow‐up (78th day of life)	< 0.6	8520

**FIGURE 1:**
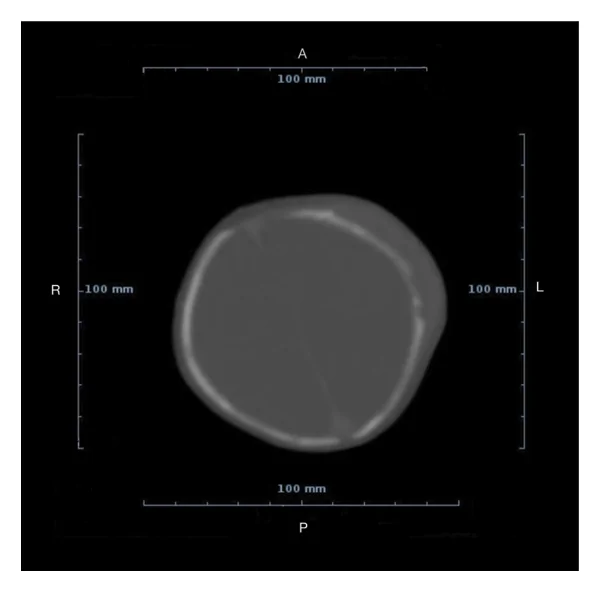
CT head with contrast (bone window); the green arrow shows the residual infected subgaleal hematoma, and the blue arrow shows the irregular thickness and reduced density of the underlying bone representing the osteomyelitis.

In the NICU, the patient remained awake, maintaining normal saturations on room air, playful, and was moving all four limbs. Broad‐spectrum antibiotics were started till the culture sensitivity was reported. Feeding was established on demand with expressed breast milk per oral, and IV fluids were tapered. On the postoperative day 1, the CRP levels dropped to 196 mg/L, WBC was 19350/μL, and the patient was shifted from the NICU to the inpatient floor, where IV antibiotics and oral feeds were continued. Serial CRP and WBCs were monitored throughout hospitalization postoperatively, demonstrating a progressive decline consistent with clinical improvement as shown in the Figure 1. The patient was closely followed by the neurosurgery and pediatrics teams. The drain was removed on the second postoperative day (34th day of life), and the dressing was changed regularly. The pus culture came out positive for MSSA. Meropenem and vancomycin were discontinued, and IV cefazolin was started. The patient remained vitally stable, with no fever spikes, tolerated oral feeds well, and passed stool and urine normally. On the fourth postoperative day, the CRP levels had fallen to 16.70 mg/L, and WBC was 20,010/μL. The patient was discharged in stable condition with an IV cannula for continuation of antibiotics (cefazolin 8 hourly) for 10 more days. She was sent home with an IV cannula instead of a PICC line, considering the unavailability of proper nursing care to tend to the PICC line, the IV line being easier to manage for the parents at home and the unanimous opinion of the medical team that an IV line would be safe and sufficient.

**FIGURE 2:**
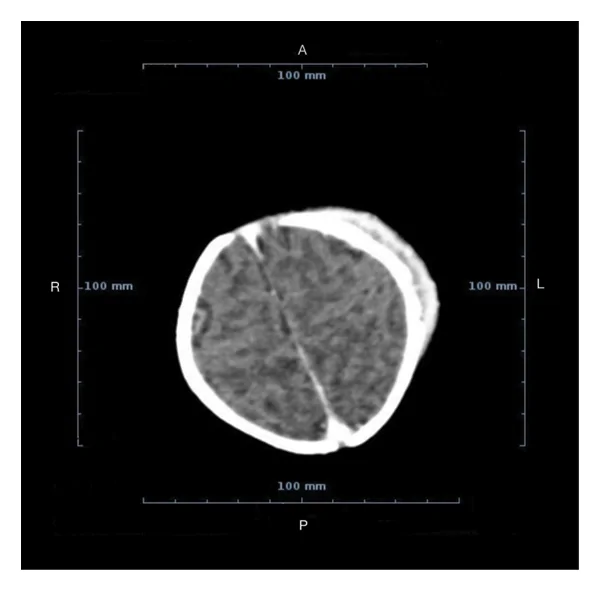
CT head with contrast (brain window); the yellow arrow shows the unaffected brain parenchyma, and the red arrow shows the irregular thickness of the infected bone underlying the infected subgaleal hematoma.

The patient returned to the emergency department of our institution 3 days later, on the 8th postoperative day (39th day of life), with excessive crying and pus discharging from the operative wound site. There was no reported fever, vomiting, seizures, or unconsciousness. On examination, the wound was mildly inflamed, with oozing from one corner. The rest of the systemic examination was unremarkable. CRP level was 5.36 mg/L, and WBC was 23260/μL, and the CT scan of the head showed a partly dense, subgaleal collection along the left frontal convexity, showing mild enhancement, consistent with residual infected subgaleal hematoma. Associated underlying bone thickening/reduced density was also noted. There was no acute intracranial hemorrhage, midline shift, mass effect, or brain herniation (Figures 1 and 2). Differentials based solely on the CT findings, other than an infected cephalhematoma, were a scalp abscess, cellulitis, or caput secundum, but correlating to the clinical features present in the patient, infected cephalhematoma was the provisional diagnosis made.

The patient underwent debridement of the infected wound site along with removal of the evidently necrotic bone (craniectomy). A pus sample was sent for culture and sensitivity, which subsequently showed only a few pus cells, likely due to prior antibiotic therapy, resulting in a culture‐negative report. The blood culture yielded negative results, actively ruling out sepsis. Postoperatively, the patient was vitally and clinically stable and active in the NICU. On the first postoperative day, the CRP levels were measured to be 3.50 mg/L, and the WBC was 38770/μL. Oral feed was well tolerated, and IV antibiotic cefazolin was started. Since the patient was now admitted to the hospital with round‐the‐clock specialized nursing care, a PICC line was considered appropriate and was established on postoperative day 2. The surgical site was examined regularly and revealed a healthy‐looking healing wound.

On the fifth postoperative day, the patient was vitally stable, active, alert, and shifted to the floor following evaluation of the wound site, which appeared clear and dry.

The PICC line was removed on the 9th postoperative day. CRP levels were 4.54 mg/L, and WBC was 10780/μL. The parents were counseled about wound care, and the patient was discharged in stable condition with continued oral antibiotics for 4 weeks.

The patient presented for follow‐up a week later at the neurosurgery and pediatrics outpatient clinics, thriving well and in stable condition. The surgical site was examined to be healing well with no signs of infection or inflammation. She was called for follow‐up again a month later in both departments, where she was noted to be recovering well, the wound site had healed completely, and there were no residual signs of infection or inflammation (CRP was 0.6 mg/L, and WBC was 8520/μL). A detailed timeline of the patient’s course of treatment is mentioned in Table 2, where trending up for the WBC is evident, most likely due to persistent localized infection of the cephalhematoma; the CRP levels were progressively decreasing under the effect of the antibiotics.

**TABLE 2:** Timeline of the patient’s treatment course.

Timeline	Subjective findings	Objective findings	Interventions
Birth	Via emergency LSCS (due to placental abruption) Uneventful discharge	None	None

5th day of life	2–3 cm lesion (no associated symptoms, thriving well)	Examined at the local hospital, clinically diagnosed as a cephalhematoma	None, counseled and sent home

25th day of life	6 cm lesion (no associated symptoms, thriving well)	USG performed at the local hospital showed 25 mL collection of fluid seen	Counseled to monitor for any changes or associated symptoms and sent home

29th day of life	7 cm lesion (no associated symptoms, thriving well)	A CT brain performed after the drainage showed residual 19 mL fluid	35 mL of pus was drained and sent for C/S (yielded *Staphylococcus aureus* a few days later) Diagnosed with infected cephalhematoma, CT brain was performed, and she was referred to a tertiary care hospital

31st day of life	Presented to our institution 7 cm lesion (no associated symptoms, thriving well)	Previous CT reviewed CRP: 207 mg/L and WBC: 18290/μL	Incision and drainage was performed, 30 mL pus aspirated and sent for C/S (yielded MSSA), a drain was placed, and the patient was admitted after the procedure in the NICU and started on antibiotics

POD # 4	Clinically well, the drain from the surgical site had been removed, dressing dry and intact	CRP was 16.70 mg/L, and WBC was 20010/μL	Discharged with an IV cannula on IV antibiotics for 10 days

POD # 8 (of first operation)	Presented to the ER with excessive crying and purulent discharge from the surgical site	CRP was 5.36 mg/L, and WBC was 23260/μL CT brain showed the cephalhematoma and underlying osteomyelitis of the parietal bone	Debridement and craniectomy were performed, and the patient was admitted to the NICU again on IV antibiotics

POD # 2 (of 2nd operation)	Dressing dry and intact Patient vitally stable Tolerating oral feeds Active and alert	PICC line inserted for antibiotic administration	None

POD # 9 of 2nd operation (day of discharge of 2nd admission)	Dressing dry and intact	CRP was 4.54 mg/L, and WBC was 10780/μL. PICC line was removed	None, the patient was discharged on oral antibiotics for the next 4 weeks

Outpatient (1 week)	Dressing dry and intact Vitally stable Active alert Tolerating oral feeds	Recovering well postoperatively	None

Outpatient (1 month)	Dressing dry and intact Vitally stable Active alert Tolerating oral feeds	CRP was < 0.6 mg/L, and WBC was 8520 μL	None

Written informed consent for publication of this case report was taken from the parents (the patient’s legal guardians), who expressed their relief and gratitude for the timely and appropriate management of the patient, while also reporting that they were satisfied with the counseling and follow‐up care that was received.

## 3. Discussion

In neonates, birth trauma to the skull can result in a cephalhematoma, defined as a hemorrhage between the bone and the periosteum [4]. The reported incidence varies in different types of deliveries, occurring in 1%–2% of spontaneous vaginal deliveries and 3%–4% of assisted deliveries (vacuum or forceps) [5]. It has also been reported after cesarean sections, as in our patient, who was born via an emergency cesarean section due to placental abruption. In most scenarios, they require no treatment and resolve spontaneously in a few weeks [2].

Acute neonatal osteomyelitis has an incidence of 1–3 per 1000 neonates in NICU settings. Patients usually present with nonspecific features, many without any reported fever, which is postulated to be due to the absence of fully developed immune systems in neonates. Owing to this, late diagnoses are made, resulting in an increased risk for long‐term consequences [6]. The vast majority of acute neonatal osteomyelitis cases are attributed to three bacteria: MSSA (26.8%), *Escherichia coli* (16.9%), and *Klebsiella pneumoniae* (14.1%). In the infant population, however, MSSA and methicillin‐resistant *S. aureus* (MRSA) account for 38.9% and 33.3%, respectively [6].

On rare occasions, cephalhematomas may acquire infections, which may be early onset, during the initial 2 weeks of life, when the etiology is either direct birth trauma or hematogenous spread, or it may take place later, during or after the third week of life, owing to cellulitis of the overlying skin [4].

As per a literature review study conducted in 2023, there are 58 reported cases of infected cephalhematomas [4]. Across the literature, *Escherichia coli* has been reported to be the pathogen responsible for a vast majority of these infections [2, 4, 5], followed by *S. aureus*, then *Enterococcus faecalis*, and *Bacteroides fragilis* [2]. The pus culture of our patient yielded MSSA, and the suspected pathway of the infection was through the skin.

Infected cephalhematomas may present as a tender, fluctuant mass, progressively increasing in size with erythema, skin abrasion, and purulent discharge. Systemic signs of infection may be present, such as pyrexia, irritability, and decreased feeding [4]. Most of these signs were not observed for the first time in our patient, the main complaint being the fluctuant swelling over the left parietal region, which had been increasing in size. The neonate had been otherwise well with no signs of local inflammation or systemic infection. The patient presented with signs of infection on the 8th POD with a mildly inflamed surgical wound and purulent discharge.

The gold standard investigation for the diagnosis of an infected cephalhematoma is needle aspiration followed by a pus culture. Baseline tests such as complete blood count, blood culture, and CRP should be measured to assess the overall status of the patient. Additionally, a cultural sensitivity of the CSF may also be performed if clinical signs pointing toward meningitis are present [7]. The baseline workup for our patient was carried out to reveal raised CRP and WBCs; however, the blood culture was negative. A CSF culture was not performed since the patient was afebrile and signs of meningeal irritation were absent. The pus culture acquired from the needle aspiration in the outside institution and from the sample obtained during the incision and drainage in our institution both yielded MSSA. It is important to note that even though aspiration is contraindicated in cephalhematomas, due to a risk of introducing microbes, it is crucial for the diagnosis of the infection and must be performed to confirm the suspicion and direct the subsequent treatment, as was the case with our patient.

A septic hematoma must be suspected when an infant begins to appear generally unwell, is febrile, or has local inflammatory features. In our case, however, the patient presented as a paradox with the sole complaint of a progressively enlarging mass and absence of any other signs or symptoms of infection (local and systemic).

Complications arising from infected cephalhematomas include sepsis, meningitis, and osteomyelitis, which is why timely diagnosis should be performed if there is a suspicion of infection [2]. A systematic review published in 2023 reported 10 cases of osteomyelitis arising as a complication secondary to infected cephalhematomas in an overall pool of 58 patients [4].

There is no specified time period for parenteral antibiotics once the causative pathogen is confirmed; however, the general recommendation is 4–6 weeks, which was the treatment protocol followed in our patient as well. A shorter course or delayed diagnosis has been associated with the development of meningitis [2]. Literature shows a great variability in how septic hematomas are managed, ranging from isolated antibiotic treatment to surgical interventions comprising either needle‐aided drainage, incision and drainage, or frank debridement [8]. When our patient first presented, the pus had been aspirated at some other hospital and was sent for culture without commencing any antibiotics. The latter course was in our institution as specified above.

Infected cephalhematomas with secondary osteomyelitis have an overall good prognosis, with most patients making complete recoveries in a few weeks without further complications, provided that timely intervention is initiated [2]. The reported mortality rate is 8.6% [4].

Through this report, we aim to contribute to the scarce body of literature by illustrating an atypical presentation of an infected cephalhematoma with secondary osteomyelitis in a neonate, where systemic and local signs were minimal. Additionally, the identification of MSSA as the causative pathogen broadens is a relatively rare occurrence and adds to the spectrum of organisms reported in such cases. This case further emphasizes evidence‐based management, combining timely surgical intervention and targeted antibiotic therapy, thereby confirming and extending current clinical knowledge.

## 4. Conclusion

An elevated index of suspicion, early diagnosis, and timely multidisciplinary care are necessary for the rare but life‐threatening illness known as infected cephalhematoma accompanied by osteomyelitis. This case report exemplifies the importance of closely monitoring infant cephalhematomas and promptly evaluating any atypical symptoms, such as persistent swelling, erythema, or systemic symptoms, to reduce the risk of potentially fatal outcomes.

## Funding

This study did not receive any funding.

## Conflicts of Interest

The authors declare no conflicts of interest.

## Data Availability

The data that support the findings of this study are available from the corresponding author upon reasonable request.
